# Three new species of the genus *Trilacuna* Tong & Li, 2007 (Araneae, Oonopidae) from China

**DOI:** 10.3897/zookeys.1283.194474

**Published:** 2026-06-25

**Authors:** Siyi Wang, Shuchong Bai, Yanfeng Tong, Dongju Bian, Shuqiang Li

**Affiliations:** 1 College of Life Science, Shenyang Normal University, Shenyang 110034, China Institute of Applied Ecology, Chinese Academy of Sciences Shenyang China; 2 College of Paleontology, Shenyang Normal University, Shenyang 110034, China College of Life Science, Shenyang Normal University Shenyang China; 3 Institute of Applied Ecology, Chinese Academy of Sciences, Shenyang 110016, China College of Paleontology, Shenyang Normal University Shenyang China; 4 Forest Canopy Biodiversity Research Center, College of Life Sciences, Anhui Normal University, Wuhu, Anhui 241000, China Forest Canopy Biodiversity Research Center, College of Life Sciences, Anhui Normal University Wuhu China

**Keywords:** Distribution, *Dysderoides* complex, morphology, oonopid spider, taxonomy

## Abstract

Three new species of the genus *Trilacuna* Tong & Li, 2007 are described from China: *T. batang* Tong & Li, **sp. nov**. (♂♀) and *T. kangding* Tong & Li, **sp. nov**. (♂♀) from Sichuan Province, and *T. shennongjia* Tong & Li, **sp. nov**. (♂♀) from Chongqing Municipality and Hubei Province. Detailed morphological descriptions, diagnostic characteristics and morphological photographs of the new species are provided.

## Introduction

The spider family Oonopidae is one of the most species-diverse spider families, with 1984 extant species placed in 115 genera (WSC 2026). The genus *Trilacuna* was established by Tong and Li (2007) to accommodate two new species from Chongqing and Yunnan, China. Subsequently, many additional species have been described: 15 from Indonesia (Sumatra), Malaysia, Myanmar, Thailand and Vietnam (Eichenberger and Kranz-Baltensperger 2011; Tong and Li 2013; Tong et al. 2020); seven from Bhutan, India, Nepal and Pakistan (Grismado et al. 2014); one from Iran (Malek-Hosseini et al. 2015); one from Korea (Seo 2017); and 32 from China (e.g., Tong et al. 2018, 2019, 2024; Liu et al. 2019; Huang et al. 2021; Wang et al. 2021; Liu et al. 2023; Ma et al. 2023, 2024; Dai et al. 2025a, 2025b, 2025c; Shi et al. 2025). To date, the genus *Trilacuna* comprises 55 valid species, all endemic to Asia (WSC 2026).

The genus *Trilacuna* Tong & Li, 2007 belongs to the “*Dysderoides* complex”, which additionally comprises three genera: *Bannana* Tong & Li, 2015, *Dysderoides* Fage, 1946 and *Himalayana* Grismado, 2014. Members of this complex share the morphology of the chelicerae, labium, and male and female genitalia (Grismado et al. 2014; Tong and Li 2015). *Bannana* is endemic to Xishuangbanna, China; *Dysderoides* is confined to India and Thailand; *Himalayana* has only been documented from Nepal and India. In contrast, *Trilacuna* possesses a much wider distribution, ranging west to Iran, east to South Korea and south to Sumatra.

In the present paper, three new species of *Trilacuna* collected from Chongqing, Hubei and Sichuan Provinces of China are reported, including two from Sichuan and one from Chongqing and Hubei. Prior to this study, only one congener, *T. guangwu* Ma & Tong, 2024, was known from Sichuan (Ma et al. 2024). Detailed morphological descriptions and diagnostic illustrations of the new species are provided herein.

## Materials and methods

The specimens were examined under a Leica M205 C stereomicroscope. Fine details were studied under an Olympus BX51 compound microscope. The vulvae were cleared in lactic acid. Photomicroscope images were taken with a Canon EOS 750D zoom digital camera (24.2 megapixels) mounted on the Olympus BX51. Raw photos were first stacked using Helicon Focus v. 8.2.0 to produce composite images, which were then processed in Adobe Photoshop CC 2020. Scanning electron microscope images (SEM) were taken under high vacuum with a Hitachi S-4800 after critical-point drying and gold-palladium coating. The distribution map was generated with ArcGIS v. 10.2 (ESRI Inc.). All measurements were taken using the Olympus BX51 and are in millimeters. Taxonomic descriptions follow Tong et al. (2020). Type material is deposited in the Shenyang Normal University (SYNU) in Shenyang, Liaoning Province, China (curator: Yanfeng Tong).

The following abbreviations are used in the text and figures: ALE = anterior lateral eyes; ap = apodeme; as = anterior sclerite; bp = basal projection; bsl = bristle-shaped lobe; glo = globular structure; ksl = knife-shaped lobe; lb = lateral branch; lsl = leaf-shaped lobes; mb = median branch; PLE = posterior lateral eyes; PME = posterior median eyes; psp = posterior spiracle; sar = sclerotized, recurved arches; tba = transverse bars; tsc = transverse sclerite; wls = wing-like structure.

## Taxonomy

### Family Oonopidae Simon, 1890

#### Genus Trilacuna

Taxon classification

Animalia

AraneaeOonopidae

Tong & Li, 2007

EC6BF4AD-5A95-5200-962C-96F490D9E41C

Trilacuna Tong & Li, 2007: 333; Grismado et al. 2014: 26.

##### Type species.

*Trilacuna rastrum* Tong & Li, 2007.

##### Diagnosis.

*Trilacuna* differs from other oonopid genera, except those of the “*Dysderoides* complex” (including *Bannana* Tong & Li, 2015, *Dysderoides* Fage, 1946, *Himalayana* Grismado, 2014, and *Trilacuna*), by the enlarged male palpal femur, the very complex embolus-conductor system, and the notched labium. Males are distinguished from *Dysderoides* by the fully developed eyes and the absence of macrosetae on legs III and IV, from *Bannana* by the normally developed eyes and usually granulated carapace, and from *Himalayana* by lacking a sharp prolateral dorsal projection on the bulb. Females differ from all three genera in having an elongate postepigastric scutum that covers nearly the entire ventral abdomen (Grismado et al. 2014; Tong et al. 2020).

##### Composition.

58 species, including three described here.

##### Distribution.

Iran to the Korean Peninsula.

#### Trilacuna batang

Taxon classification

Animalia

AraneaeOonopidae

Tong & Li sp. nov.

5D00DE0D-61F7-540C-8740-2B56D54A3CE3

https://zoobank.org/FA4E525E-DAAA-4322-A130-F86FF85202D9

**Figure 1.**
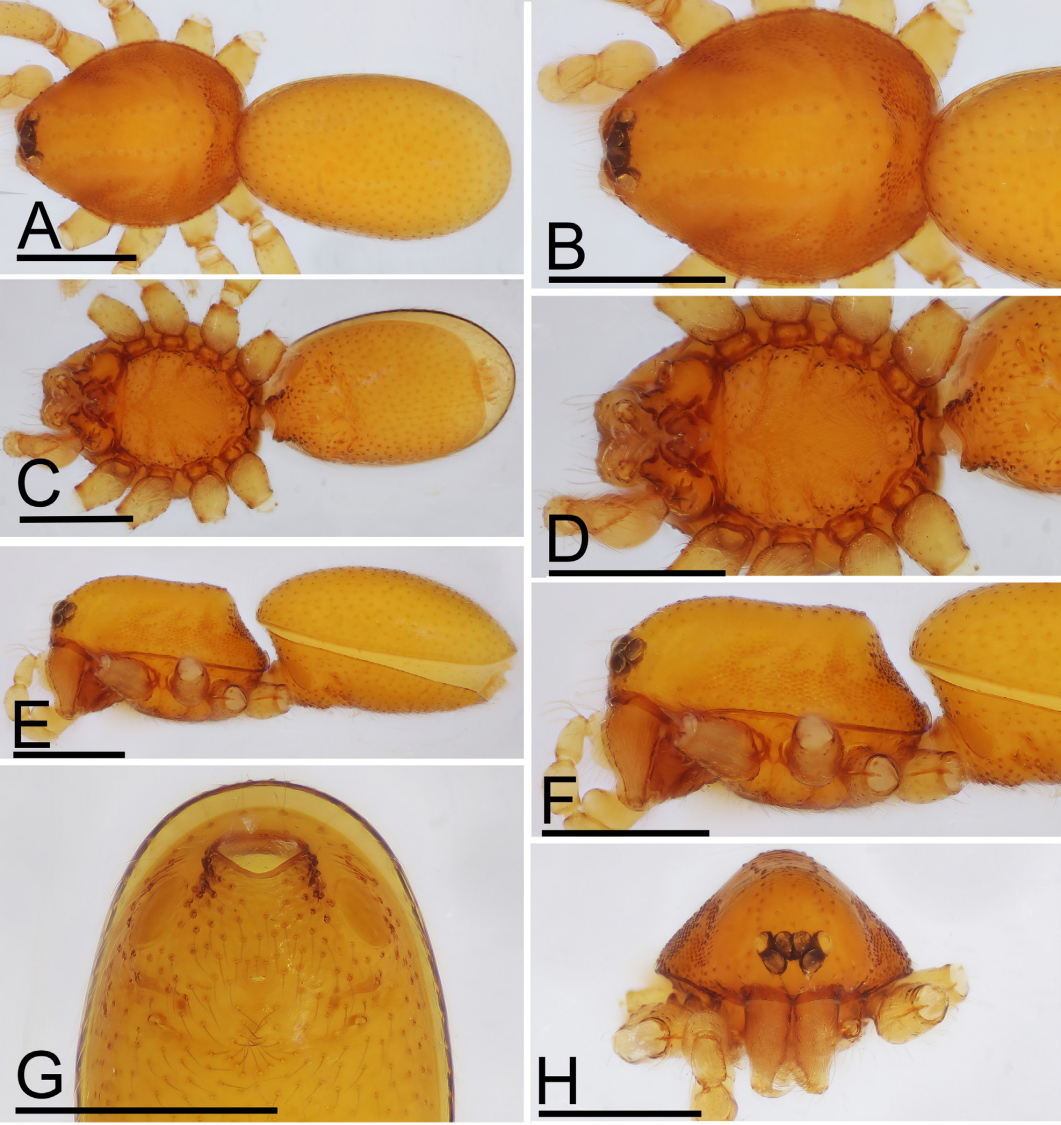
*Trilacuna batang* sp. nov., male holotype. **A, C, E**. Habitus, dorsal, ventral and lateral views; **B, D, F, H**. Cephalothorax, dorsal, ventral, lateral and anterior views; **G**. Abdomen, ventral view. Scale bars: 0.4 mm.

**Figure 2.**
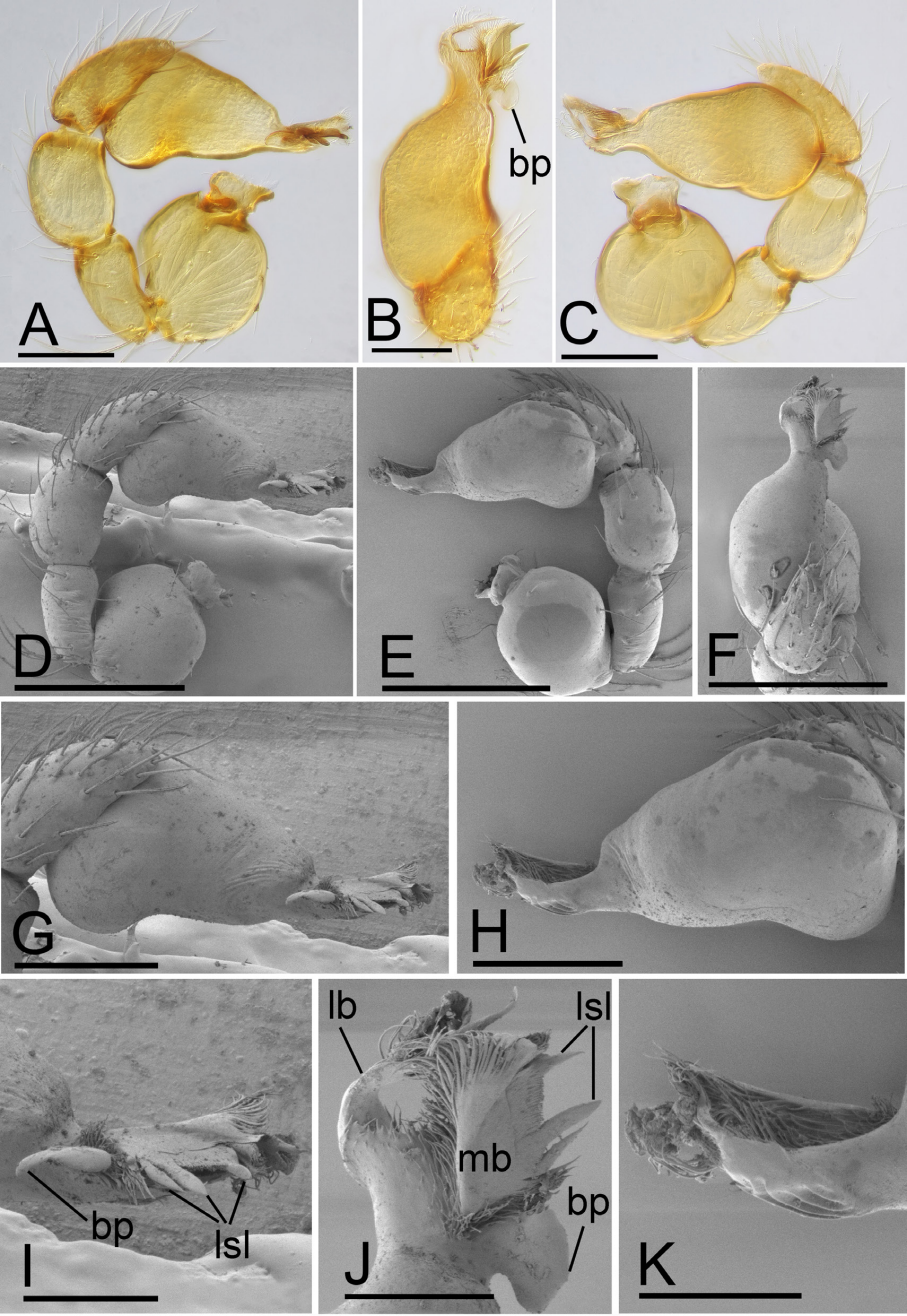
*Trilacuna batang* sp. nov., male left palp. **A, D**. Prolateral views; **B, F**. Dorsal views; **C, E**. Retrolateral views; **G, H**. Bulb, prolateral and retrolateral views; **I–K**. Distal part of bulb, prolateral, dorsal and retrolateral views. Abbreviations: bp = basal projection; lb = lateral branch; lsl = leaf-shaped lobes; mb = median branch. Scale bars: 0.1 mm (**A–C**, **G–H**); 0.2 mm (**D–F**); 0.05 mm (**I–K**).

**Figure 3.**
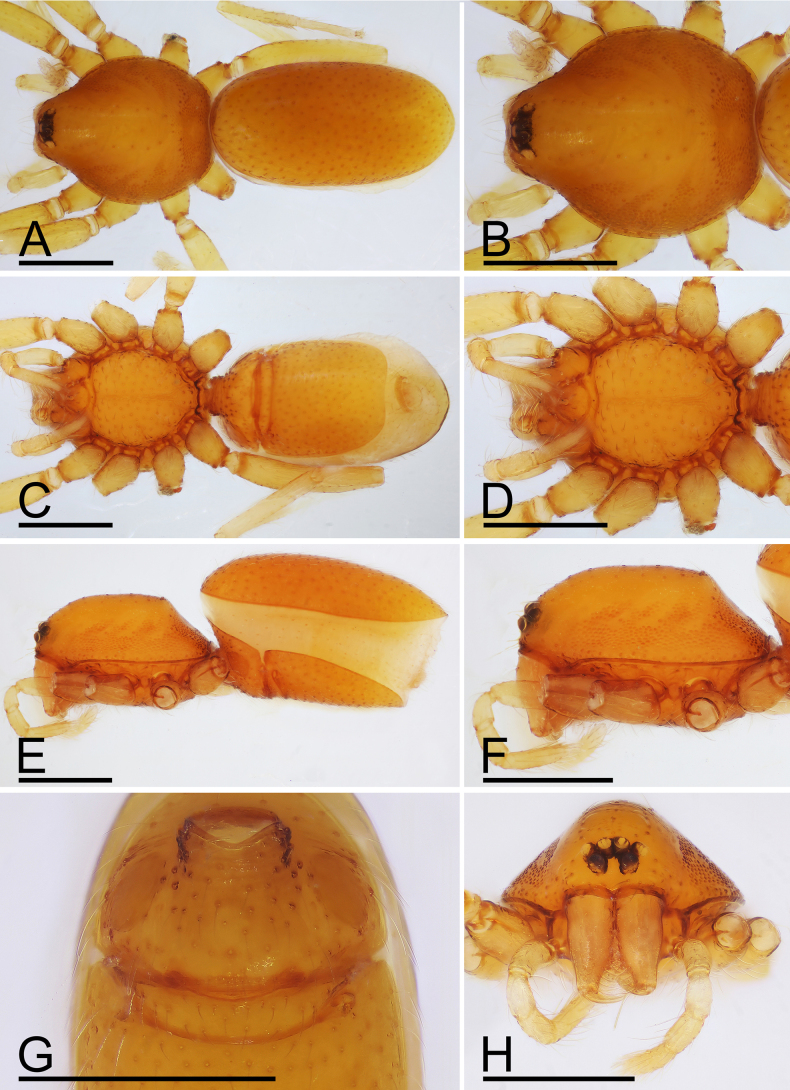
*Trilacuna batang* sp. nov., female paratype. **A, C, E**. Habitus, dorsal, ventral and lateral views; **B, D, F, H**. Cephalothorax, dorsal, ventral, lateral and anterior views; **G**. Abdomen, ventral view. Scale bars: 0.4 mm.

Figs 1, 2, 3, 10A, B, G

##### Common name.

巴塘三窝蛛.

##### Type material.

***Holotype*** China • ♂ (SYNU-903); Sichuan Prov., Batang Co., Zhubalong Town, Sangdagou; 29°59'2.40"N, 99°7'37.20"E, 2500 m elev.; 3.VI.2009; leg. Hui Zhai. ***Paratypes*** China • 2♀ (SYNU-904–905); same data as holotype.

##### Etymology.

The specific name is a noun in apposition taken from the type locality.

##### Diagnosis.

The new species is similar to *T. gongshan* Tong, Zhang & Li, 2019 in the granulate carapace and the triangular bulb, but can be distinguished by the leaf-shaped lobes of the psembolus (basal two lobes widely separated from the distal one, vs. three closely adjacent tooth-like lobes; cf. Fig. 2I, J and Tong et al. 2019: fig. 11B, D), the palpal femur nearly globular (vs. width/length = 0.6; cf. Fig. 2A, D, E and Tong et al. 2019: figs 11A, 23A) and the recurved, strongly sclerotized arches of epigastric area (vs. weakly sclerotized; cf. Figs 3G, 10Aand Tong et al. 2019: fig. 24G).

##### Description.

**Male. *Body***: yellow, legs pale yellow; habitus as in Fig. 1A, C, E; body length 1.67. ***Carapace*** (Fig. 1B, F): 0.78 long, 0.64 wide; lateral sides granulate. ***Eyes*** (Fig. 1B, H): well developed; ALE largest, PLE smallest; posterior eye row recurved from above, straight from front; ALE separated from edge of carapace by 1.1 diameters. ***Mouthparts*** (Figs 1D, 10G): endites distally branched. ***Sternum*** (Fig. 1D): surface smooth, medial area strongly rugose. ***Abdomen*** (Fig. 1A, C, E, G): 0.93 long, 0.55 wide; sperm pore situated in front of anterior spiracles; apodemes present, posterior spiracles connected by shallow groove. ***Palp*** (Fig. 2A–K): yellowish; 0.53 long (0.15, 0.12, 0.12, 0.14); femur globular (width/length = 1.0); patella as long as tibia; cymbium about 1.2 times the length of tibia; bulb triangular; psembolus complex, with basal projection (bp), leaf-shaped lobes (lsl), broad median branch (mb) and lateral branch (lb), surrounded by numerous fiber structures.

**Female**. Same as male except as noted. Body length 1.81; habitus as in Fig. 3A, C, E. Carapace 0.77 long, 0.64 wide (Fig. 3B, D, F, H). ***Abdomen***: 1.05 long, 0.55 wide. ***Epigastric area*** (Figs 3G, 10A): with recurved, strongly sclerotized arches (sar). ***Vulva*** (Fig. 10B): with narrow, curved transverse sclerite (tsc); with anterior slender stick-shaped sclerite (as) and posterior small globular structure (glo); transverse bars (tba) with pair of short lateral apodemes (ap).

##### Distribution.

Known only from the type locality (Fig. 11).

#### Trilacuna kangding

Taxon classification

Animalia

AraneaeOonopidae

Tong & Li sp. nov.

B8A9F32A-3F49-5C2B-AF9F-B8E36B22910E

https://zoobank.org/A51B3BB9-1FF1-42A2-BCB7-CA55340D352A

**Figure 4.**
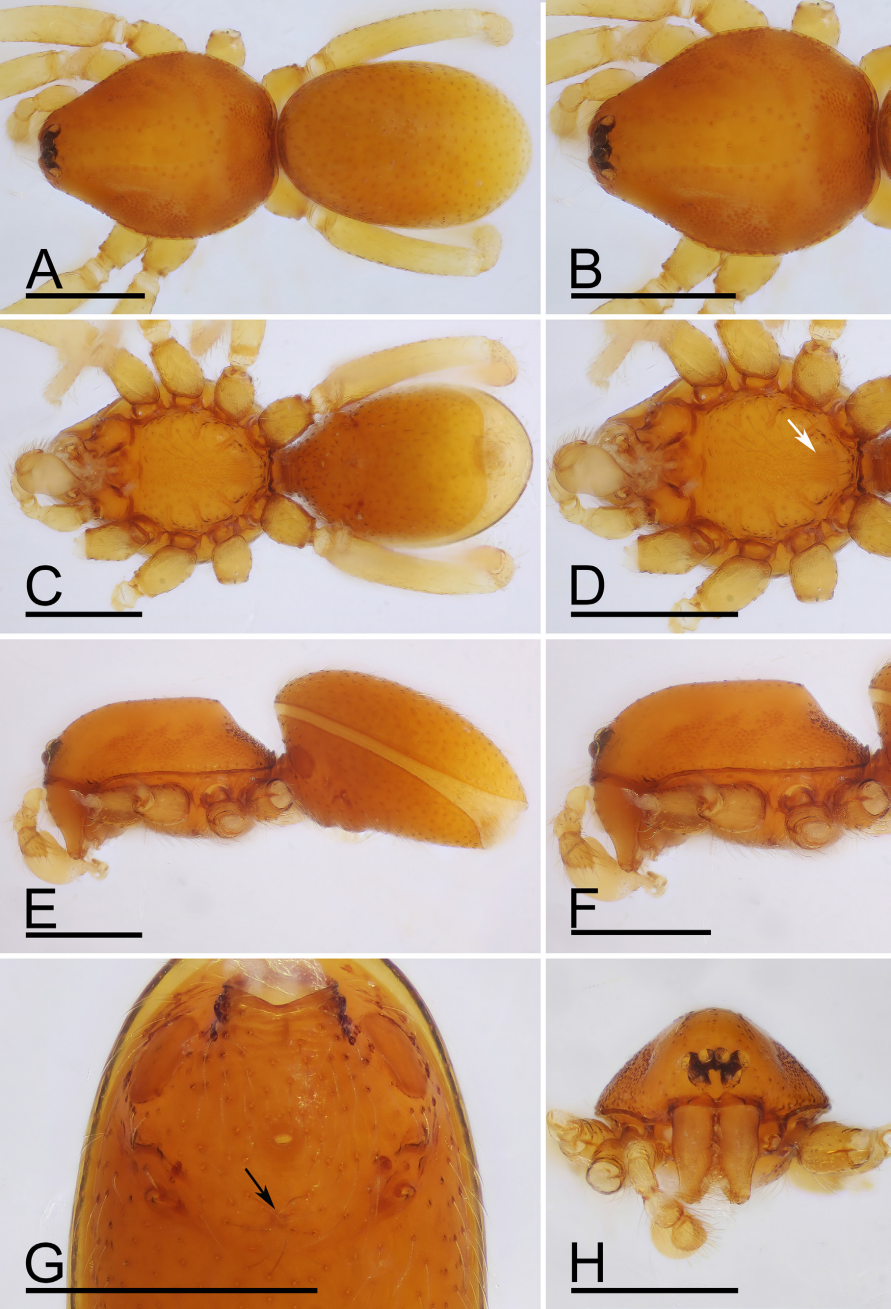
*Trilacuna kangding* sp. nov., male holotype. **A, C, E**. Habitus, dorsal, ventral and lateral views; **B, D, F, H**. Cephalothorax, dorsal, ventral, lateral and anterior views; white arrow shows the rows of ridges; **G**. Abdomen, ventral view; black arrow shows the small spot. Scale bars: 0.4 mm.

**Figure 5.**
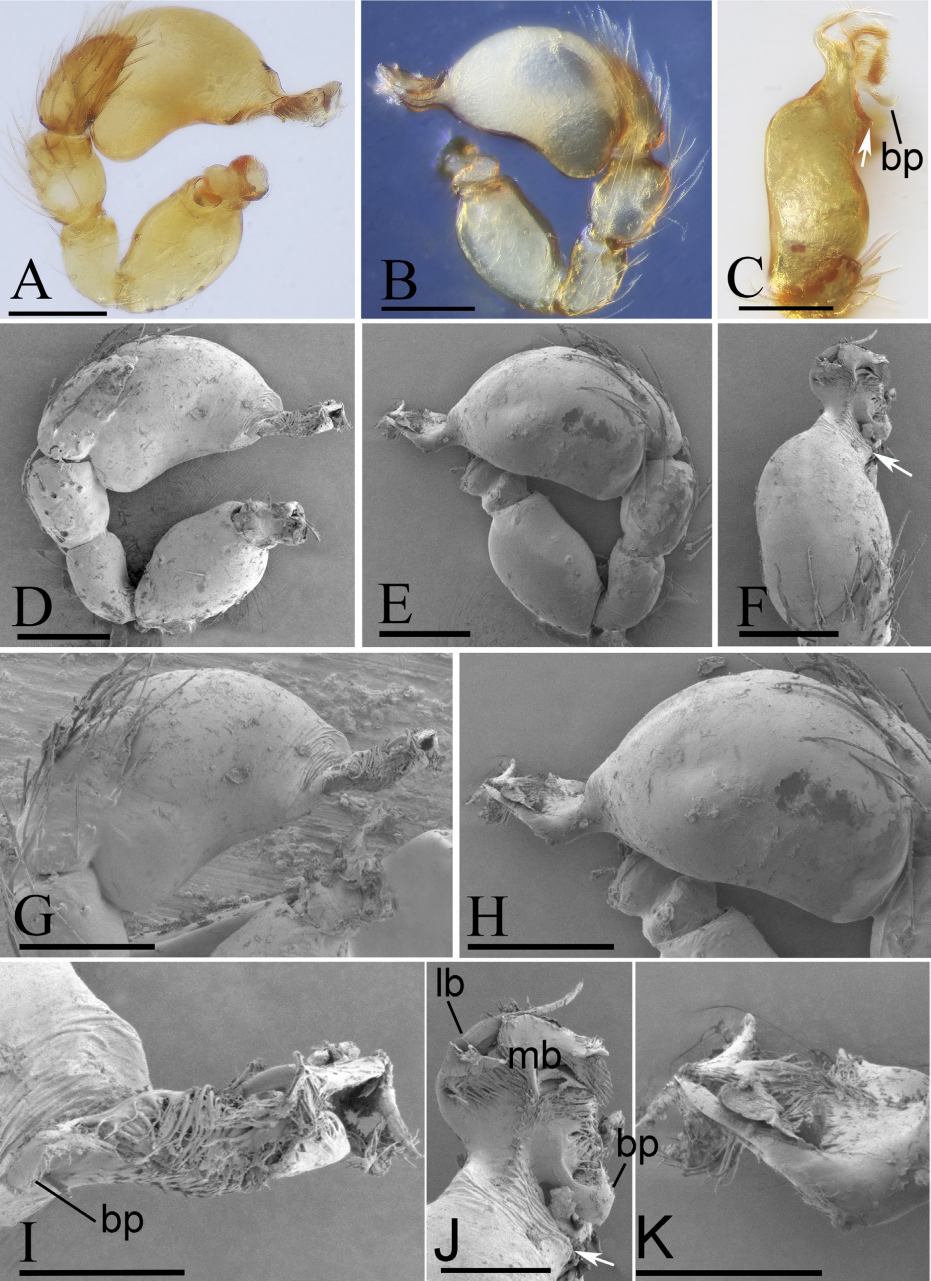
*Trilacuna kangding* sp. nov., male left palp. **A, D**. Prolateral views; **B, E**. Retrolateral views; **C, F**. Dorsal views; arrows show the apophysis; **G, H**. Bulb, prolateral and retrolateral views; **I–K**. Distal part of bulb, prolateral, dorsal and retrolateral views; arrow shows the apophysis. Abbreviations: bp = basal projection; lb = lateral branch; mb = median branch. Scale bars: 0.1 mm (**A–H**); 0.05 mm (**I–K**).

**Figure 6.**
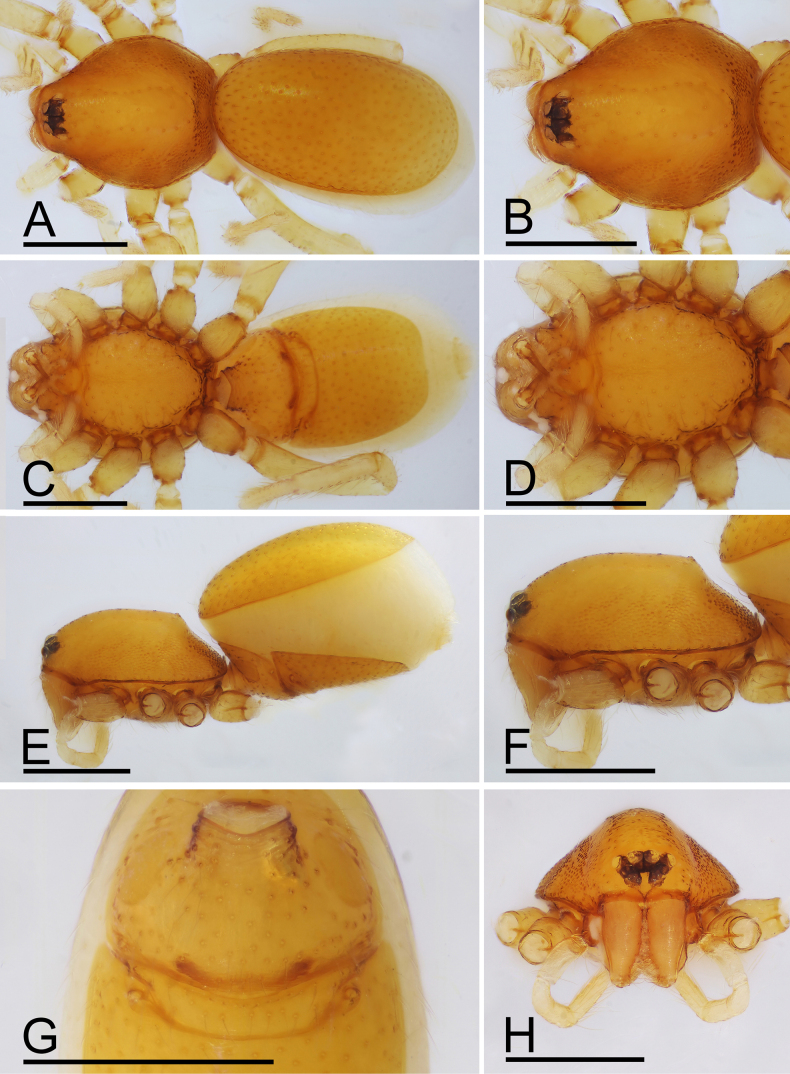
*Trilacuna kangding* sp. nov., female paratype. **A, C, E**. Habitus, dorsal, ventral and lateral views; **B, D, F, H**. Cephalothorax, dorsal, ventral, lateral and anterior views; **G**. Abdomen, ventral view. Scale bars: 0.4 mm.

Figs 4, 5, 6, 10C, D, H

##### Common name.

康定三窝蛛.

##### Type material.

***Holotype*** China • ♂ (SYNU-927); Sichuan Prov., Kangding, Shelian Township; 30°13'1.20"N, 102°11'56.40"E, 1472 m elev.; 21.V.2009; leg. Hui Zhai. ***Paratypes*** China • 1♀ (SYNU-928); same data as holotype • 1♀ (SYNU-907); Sichuan Prov., Luding Coun., west of Moxi Town, banks of Dadu River; 29°38'32.6"N, 102°7'18.89"E; 20.V.2009; leg. Hui Zhai.

##### Etymology.

The specific name is a noun in apposition taken from the type locality.

##### Diagnosis.

The new species is similar to *T. jimengi* Dai & Tong, 2025 in the kidney-shaped bulb and the small apophysis on subapical region of the bulb, but can be distinguished by the elongated palpal femur (width/length = 0.67 vs. 0.75; cf. Fig. 5A, D and Dai et al. 2025c: fig. 2A, D), the granulate carapace (vs. smooth; cf. Figs 4B, 4F, 6B, 6Fand Dai et al. 2025c: figs 1B, F, 3B, F), the rows of ridges on posterior part of sternum (vs. on median part; cf. Fig. 4D and Dai et al. 2025c: fig. 1D), and the smooth anterior margin of the postepigastric scutum (vs. with a triangular notch; cf. Figs 6G, 10Cand Dai et al. 2025c: figs 3G, 7a).

##### Description.

**Male. *Body***: yellow, legs pale yellow; habitus as in Fig. 4A, C, E; body length 1.71. ***Carapace*** (Fig. 4B, F): 0.81 long, 0.64 wide; lateral sides granulate. ***Eyes*** (Fig. 4B, H): well developed; ALE largest, PLE smallest; posterior eye row recurved from above, procurved from front; ALE separated from edge of carapace by 1.0 diameters. ***Mouthparts*** (Figs 4D, 10H): endites distally branched. ***Sternum*** (Fig. 4D): surface smooth, medial area strongly rugose; with several rows of ridges on posterior part. ***Abdomen*** (Fig. 4A, C, E, G): 0.87 long, 0.57 wide; sperm pore situated in front of anterior spiracles; apodemes present, posterior spiracles connected by shallow groove; with small spot situated between posterior spiracles. ***Palp*** (Fig. 5A–K): yellow; 0.51 long (0.16, 0.11, 0.10, 0.14); femur elongated (width/length = 0.67); patella subequal to tibia; cymbium about 1.4 times the length of tibia; bulb kidney-shape, tapering apically, with a small apophysis on subapical region (arrows in Fig. 5C, F, J); psembolus complex, with basal projection (bp), broad median branch (mb) and lateral branch (lb), surrounded by numerous fiber structures.

**Female**. Same as male except as noted. Body length 1.72; habitus as in Fig. 6A, C, E. Carapace 0.69 long, 0.59 wide (Fig. 6B, D, F, H). ***Abdomen***: 0.99 long, 0.59 wide. ***Epigastric area*** (Figs 6G, 10C): with recurved, strongly sclerotized arches (sar). ***Vulva*** (Fig. 10D): with narrow, curved transverse sclerite (tsc); with anterior slender stick-shaped sclerite (as) and posterior small globular structure (glo); transverse bars (tba) with pair of short lateral apodemes (ap).

##### Distribution.

Known only from the type locality (Fig. 11).

#### Trilacuna shennongjia

Taxon classification

Animalia

AraneaeOonopidae

Tong & Li sp. nov.

37B45D4E-E4C1-5DBA-B50F-70B70A6C090F

https://zoobank.org/C8F15CD2-D90B-4D7E-9654-48D83AE5EA0D

**Figure 7.**
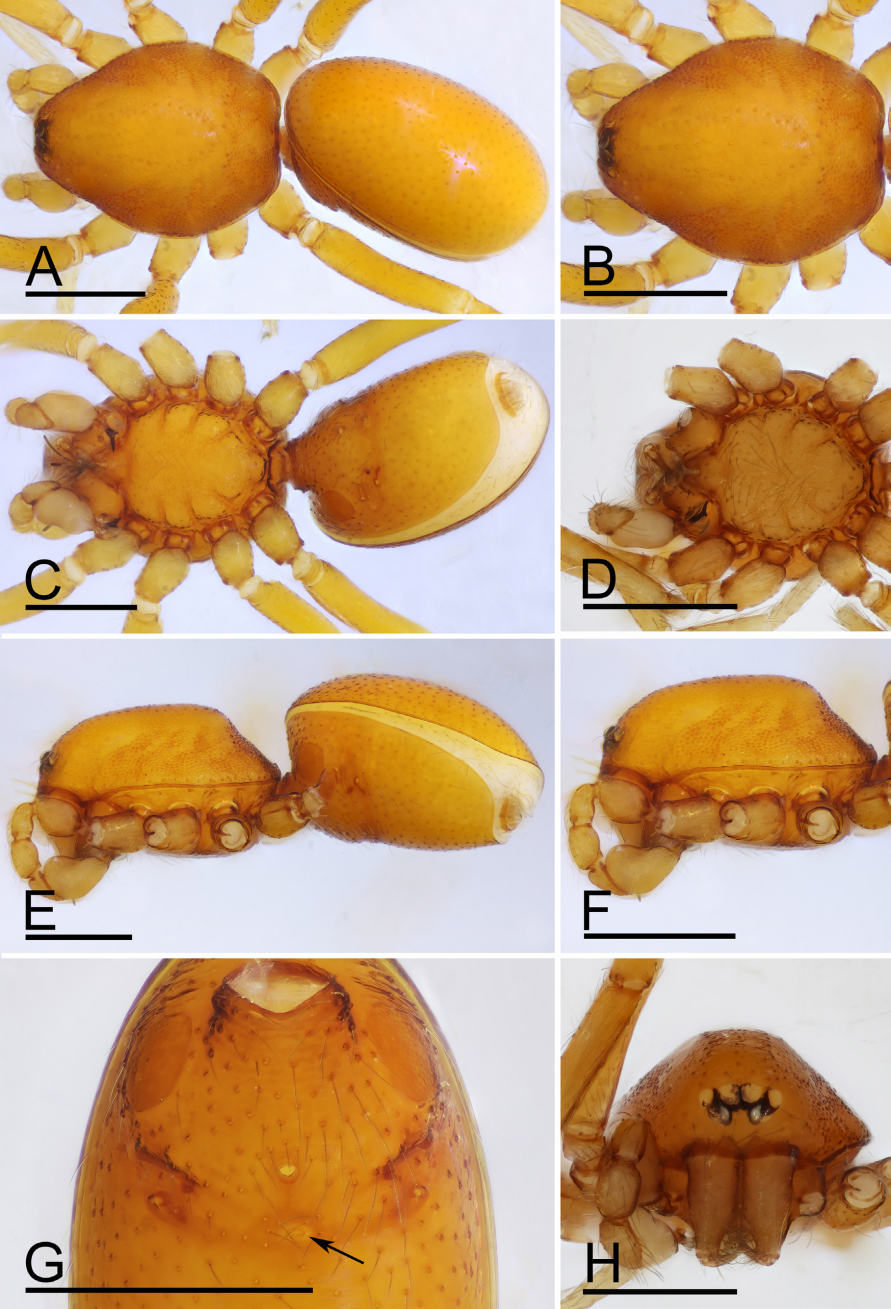
*Trilacuna shennongjia* sp. nov. **A–C, E–G**. (male holotype); **D, H**. (paratype (SYNU-1609)). **A, C, E**. Habitus, dorsal, ventral and lateral views; **B, D, F, H**. Cephalothorax, dorsal, ventral, lateral and anterior views; **G**. Abdomen, ventral view; arrow shows the small hole. Scale bars: 0.4 mm.

**Figure 8.**
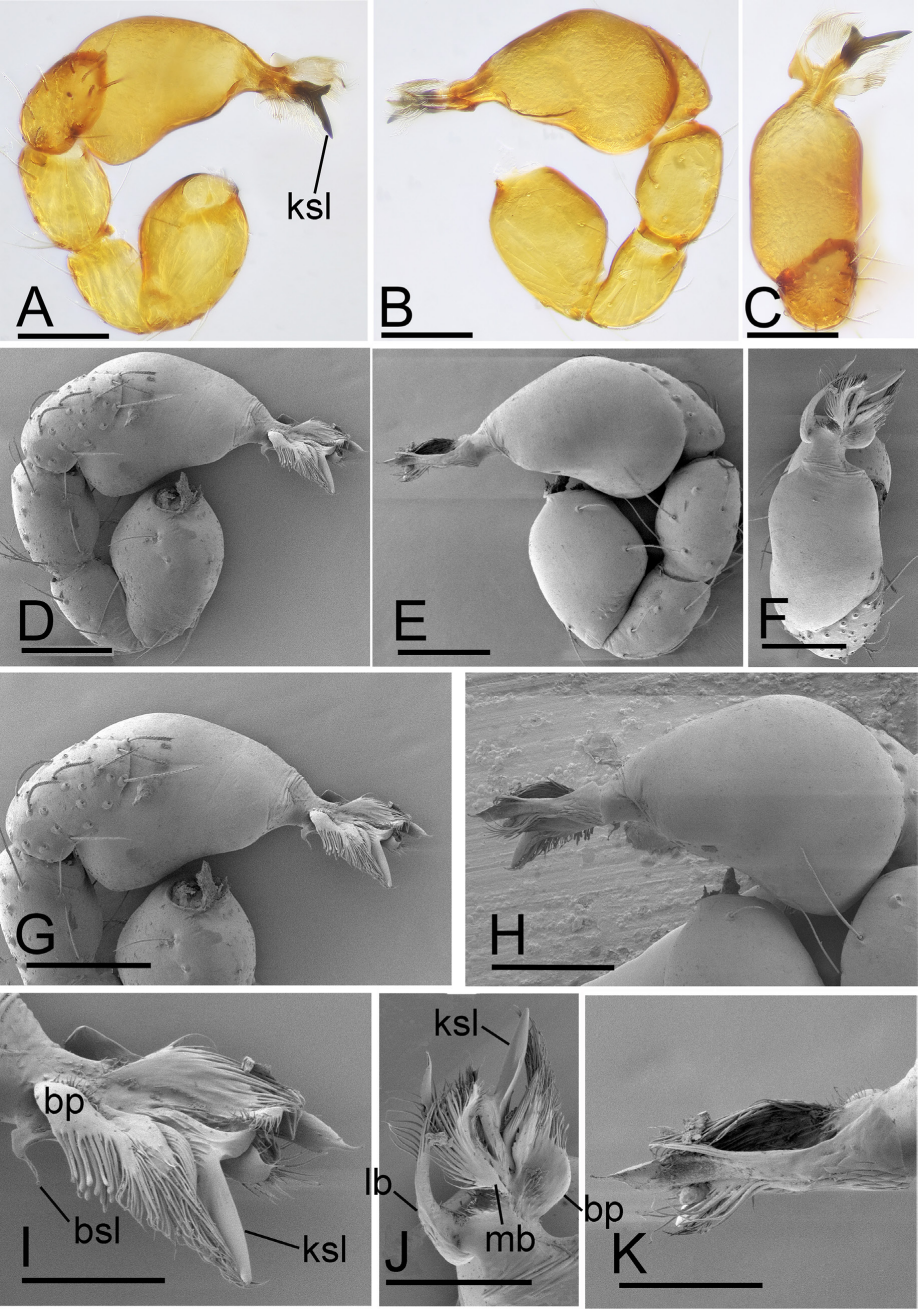
*Trilacuna shennongjia* sp. nov., male left palp. **A, D**. Prolateral views; **B, E**. Retrolateral views; **C, F**. Dorsal views; **G, H**. Bulb, prolateral and retrolateral views; **I–K**. Distal part of bulb, prolateral, dorsal and retrolateral views. Abbreviations: bp = basal projection; bsl = bristle-shaped lobe; ksl = knife-shaped lobe; lb = lateral branch; mb = median branch. Scale bars: 0.1 mm (**A–H**); 0.05 mm (**I–K**).

**Figure 9.**
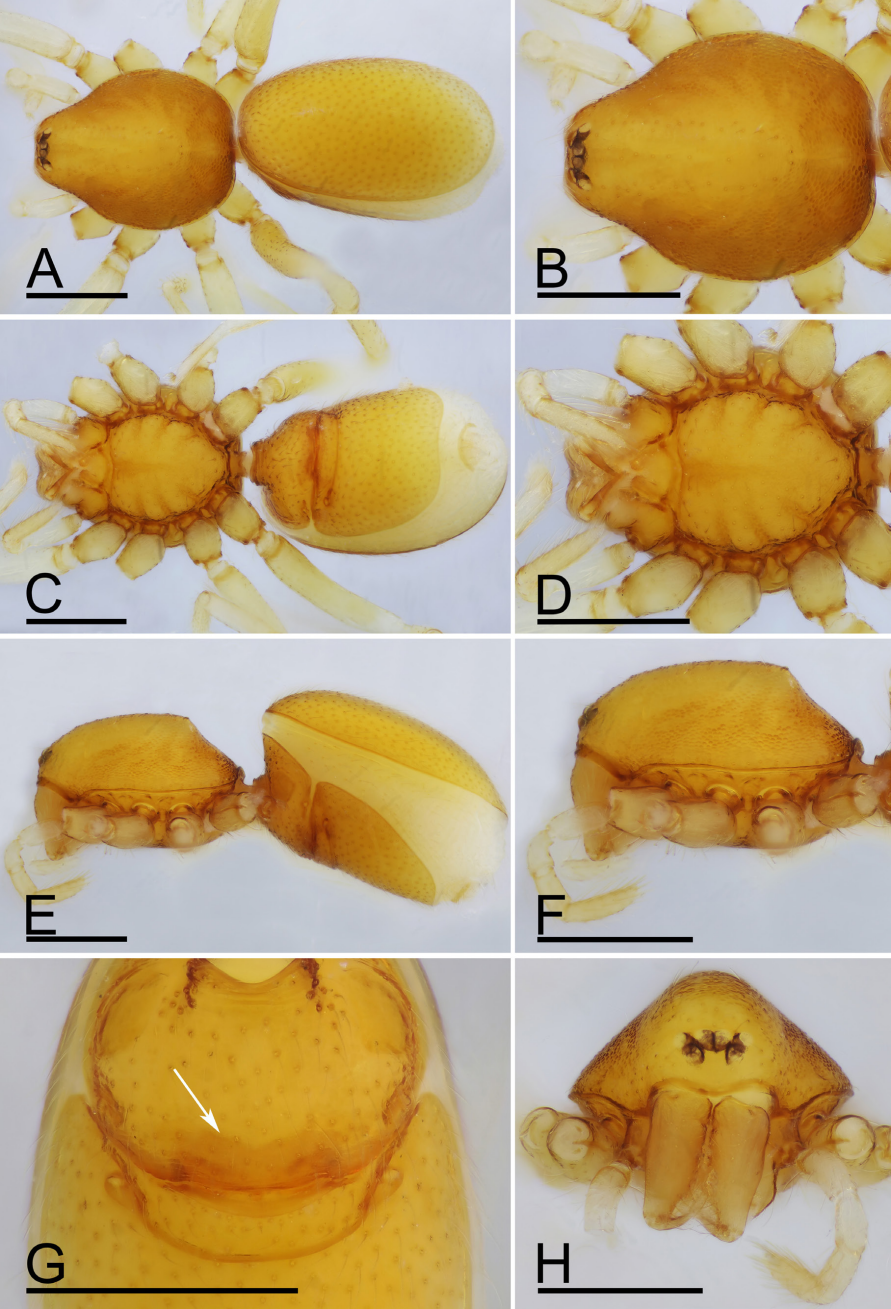
*Trilacuna shennongjia* sp. nov., female paratype. **A, C, E**. Habitus, dorsal, ventral and lateral views; **B, D, F, H**. Cephalothorax, dorsal, ventral, lateral and anterior views; **G**. Abdomen, ventral view; arrow shows the wing-like structure. Scale bars: 0.4 mm.

**Figure 10.**
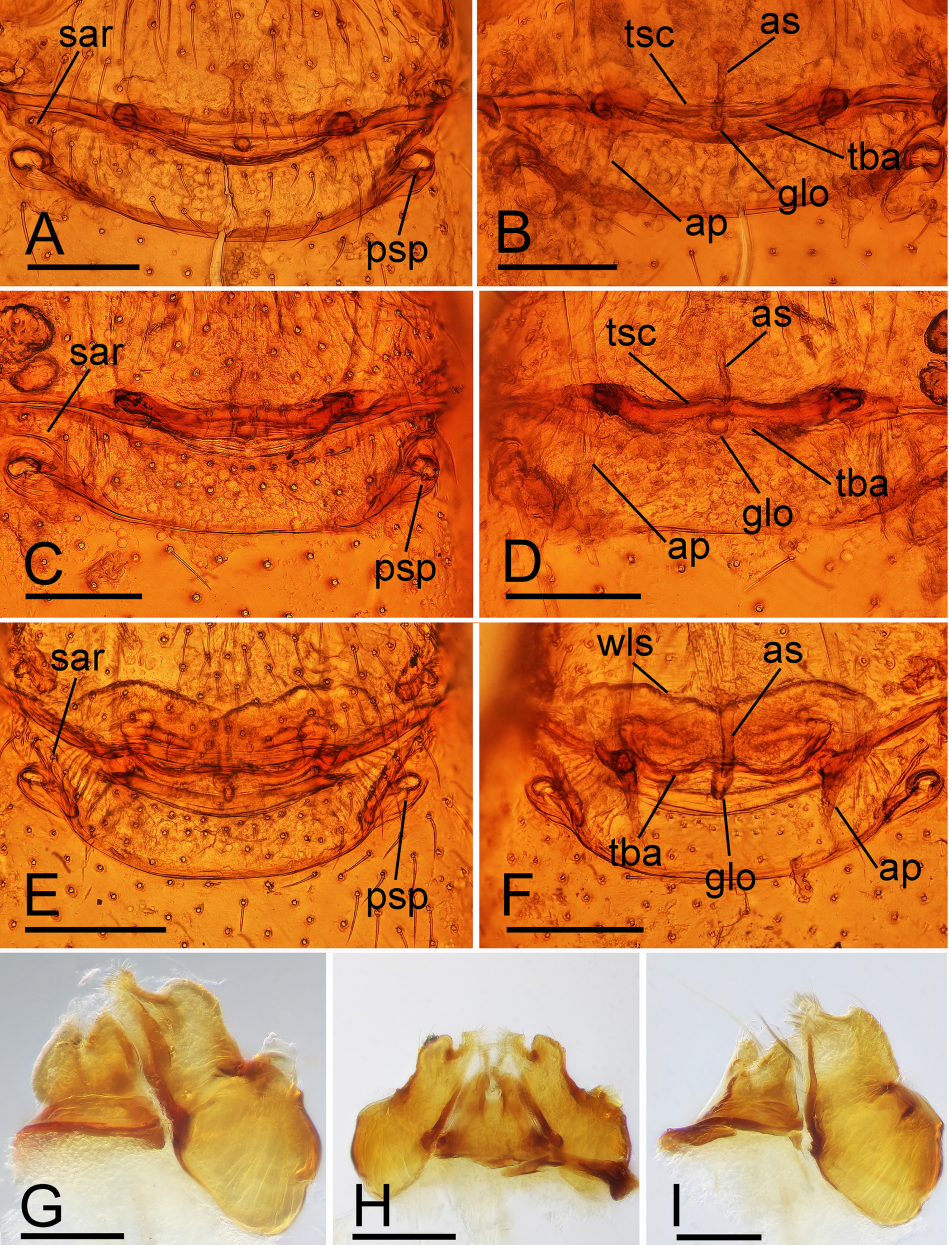
*Trilacuna* spp. **A–F**. Vulvae; **G–I**. Male labium and endites. **A, B**. *Trilacuna batang* sp. nov.; **C, D**. *Trilacuna kangding* sp. nov.; **E, F**. *Trilacuna shennongjia* sp. nov.; **A, C, E, G–I**. Ventral views; **B, D, F**. Dorsal views. Abbreviations: ap = apodeme; as = anterior sclerite; glo = globular structure; psp = posterior spiracle; sar = sclerotized, recurved arches; tba = transverse bars; tsc = transverse sclerite; wls = wing-like structure. Scale bars: 0.1 mm.

Figs 7, 8, 9, 10E, F, I

##### Common name.

神农架三窝蛛.

##### Type material.

***Holotype*** China • ♂ (SYNU-900); Hubei Prov., Shennongjia Forestry District, Muyu Town, near Qingtian Ancient Tree; 31°29'40.92"N, 110°22'48.59"E, 1432 ± 8 m elev.; 19.VII.2015; leg. H. Yang & H. Liu. ***Paratypes*** China • 1♀ (SYNU-901), Hubei Prov., Shennongjia Forestry District, Muyu Town, Guanmen Mt.; 31°26'5.611"N, 110°22'45.8"E, 1240 m elev.; 23.VII.1998; leg. Junjian He • 1♂2♀ (SYNU-1606–08), Chongqing, Chengkou Coun., Heyu Town, Xumu Vill.; 31°53'56"N, 109°2'21"E, 1475 m elev.; 16.V.2025; leg. Wang & Lu • 2♂4♀ (SYNU-1609–14), Chongqing, Chengkou Coun., Dong’an Town, Chaoyang Vill.; 31°47'28"N, 109°14'9"E, 1578 m elev.; 15.V.2025; leg. Wang & Lu • 1♂ (SYNU-1615), Chongqing, Chengkou Coun., Lianfeng Vill., Nacai valley; 32°3'0.6540"N, 108°42'44.9580"E, 1284 m elev.; 4.V.2025; leg. Wang et al. • 1♂2♀(SYNU-1616–18), Chongqing, Wuxi Coun., Shuangyang Town, Yintiaoling Natural Reserve, Luomadian, Huanglianchang valley; 31°32'31"N, 109°51'1"E, 1495 m elev.; 12.V.2025; leg. Wang & Lu.

##### Etymology.

The specific name is a noun in apposition, derived from Shennongjia, where the holotype was collected.

##### Diagnosis.

The new species is similar to *T. rastrum* Tong & Li, 2007 in the granulate carapace and the enlarged bulb, but can be distinguished by the strongly blackened knife-shaped lobe of the bulb (vs. lacking, but with a rake-shaped protuberance; cf. Fig. 8A, I and Tong and Li 2007: figs 7–10) and the wing-like structure of the vulva (vs. lacking; cf. Figs 9G, 10E, 10Fand Tong and Li 2007: fig. 6).

##### Description.

**Male. *Body***: yellow, legs pale yellow; habitus as in Fig. 7A, C, E; body length 1.73. ***Carapace*** (Fig. 7B, F): 0.83 long, 0.66 wide; lateral sides granulate. ***Eyes*** (Fig. 7B, H): well developed; ALE and PME nearly equal size, PLE smallest; posterior eye row recurved from above, procurved from front; ALE separated from edge of carapace by 1.2 diameters. ***Mouthparts*** (Figs 7D, 10I): endites distally slightly branched. ***Sternum*** (Fig. 7D): surface rugose. ***Abdomen*** (Fig. 7A, C, E, G): 0.97 long, 0.61 wide; sperm pore situated in front of anterior spiracles; apodemes present, posterior spiracles connected by shallow groove; with small hole situated between posterior spiracles. ***Palp*** (Fig. 8A–K): yellow; 0.54 long (0.17, 0.12, 0.12, 0.13); femur swollen (width/length = 0.73); patella as long as tibia; cymbium about 1.1 times the length of tibia; bulb oval, stout, tapering apically; psembolus complex, with basal bristle-shaped lobe (bsl), basal projection (bp), strongly blackened knife-shaped lobe (ksl), broad median branch (mb) and lateral branch (lb), surrounded by numerous fiber structures.

**Female**. Same as male except as noted. Body length 1.88; habitus as in Fig. 9A, C, E. Carapace 0.81 long, 0.65 wide (Fig. 9B, D, F, H). ***Abdomen***: 1.05 long, 0.63 wide. ***Epigastric area*** (Figs 9G, 10E): with recurved, strongly sclerotized arches (sar); wing-like structure can be seen through the cuticle. ***Vulva*** (Fig. 10F): with membranous, wing-like structure (wls); with anterior slender stick-shaped sclerite (as) and posterior small globular structure (glo); transverse bars (tba) with pair of short lateral apodemes (ap).

##### Distribution.

**Figure 11.**
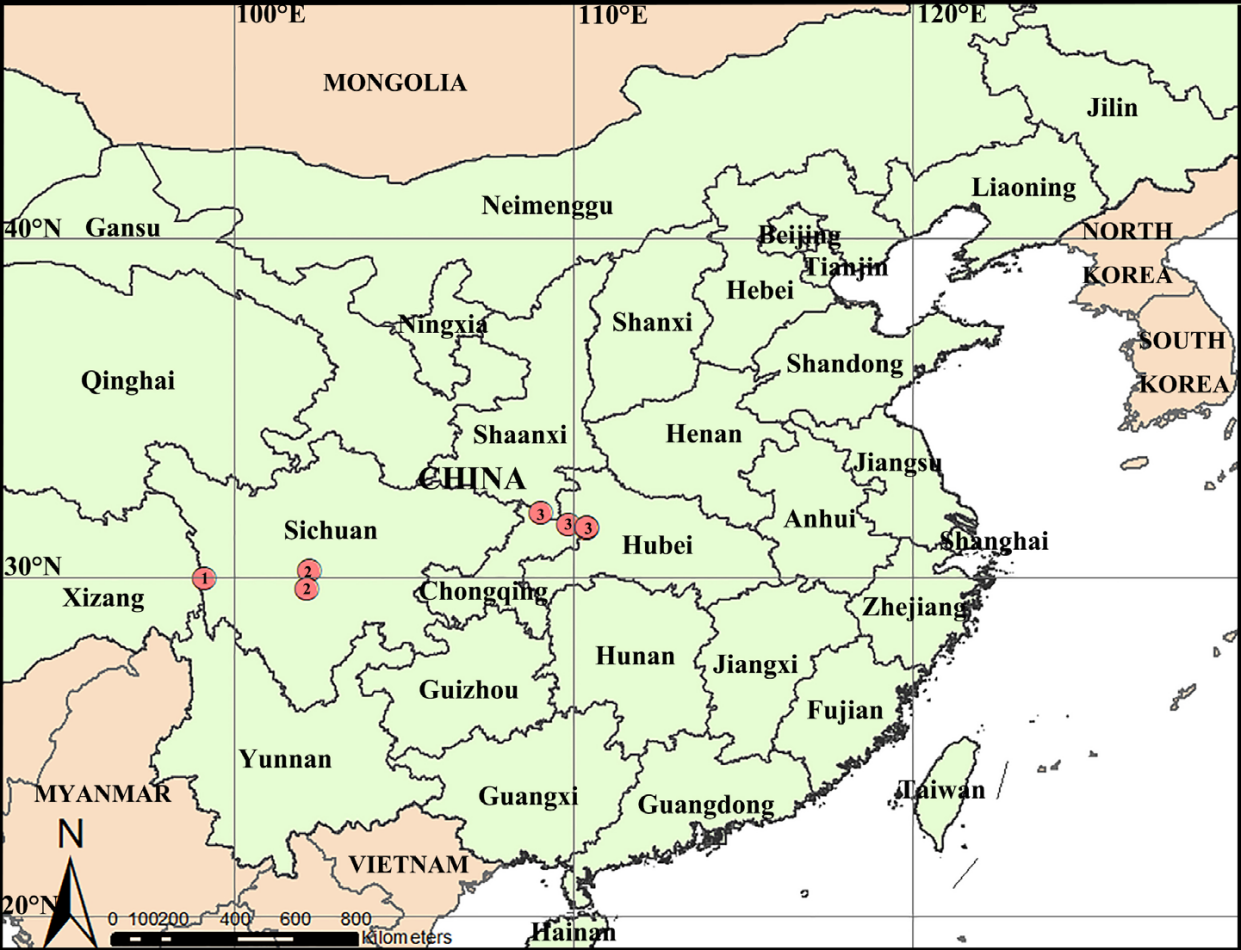
Distribution records of three new species from China. 1. *Trilacuna batang* sp. nov.; 2. *Trilacuna kangding* sp. nov.; 3. *Trilacuna shennongjia* sp. nov.

Known only from the type locality (Fig. 11).

## Supplementary Material

XML Treatment for Trilacuna

XML Treatment for Trilacuna batang

XML Treatment for Trilacuna kangding

XML Treatment for Trilacuna shennongjia

## Additional information

### Artificial Intelligence (AI) use

The authors accept full responsibility for the content of the manuscript, including the disclosure of any use of AI.

The authors used Doubao for language refinement in the manuscript.

### Author ORCIDs

S. Wang https://orcid.org/0009-0004-3858-0693

S. Bai https://orcid.org/0000-0001-7624-5483

Y. Tong https://orcid.org/0000-0002-4348-7029

D. Bian https://orcid.org/0000-0002-9324-8026

S. Li https://orcid.org/0000-0002-3290-5416

### Data availability

All of the data that support the findings of this study are available in the main text.
